# Cell membrane breakage and triggering T cell infiltration are involved in human telomerase reverse transcriptase (hTERT) promoter-driven novel peptide KK-64 for liver cancer gene therapy

**DOI:** 10.1080/21655979.2021.2010314

**Published:** 2021-12-14

**Authors:** Yuanhua Lu, Jie Ma, Jian Lin, Yafei Tian, Yongjun Ma, Wei Wang, Jialin Li, Hugang Zhang, Ping Jiao

**Affiliations:** School of Pharmaceutical Sciences, Jilin University, Changchun, China

**Keywords:** ACP, liver cancer, KK-64, apoptosis, tumor growth

## Abstract

Liver cancer is an aggressive malignancy with exhibits both high mortality and morbidity. The current treatment options are associated with several limitations, novel specific anti-cancer drugs are urgently needed to improve liver cancer treatment. In this study, a new peptide KK-64 was designed, and it showed strong cytotoxicity against liver cancer cells. To obtain the tumor targeting property, a plasmid that contains KK-64 DNA fragment and driven by human telomerase reverse transcriptase (hTERT) promoter was constructed. pcTERT-kk-64 plasmid was found to specifically inhibit the viability of liver cancer cells HepG2, induce substantial apoptosis as well as damage to the cell membranes, but had minimal effects toward normal liver HL-7702 cells. Furthermore, pcTERT-kk-64 plasmids was also noted to significantly attenuate migration and invasion of HepG2 cells. The anti-tumor effect of pcTERT-kk-64 plasmid was also observed in H22 cell-bearing mice, and it appeared to cause significant tumor regression, trigger tumor cell apoptosis, and infiltrate cytotoxicity T cells to the tumor tissues after plasmids injection. Thus, pcTERT-kk-64 plasmids showed both strong cytotoxicity and tumor selectivity in vitro and in tumor-bearing mice in liver cancer models.

## Introduction

Liver cancer is a lethal disease affecting human health, and its incidence and mortality have been reported to be in the top 10 of cancers worldwide [1]. Despite an improvement in the field of liver cancer therapy, multiple obstacles have limited the therapeutic progress [2–4]. Efficient strategies are needed for liver cancer therapy. Presently, anticancer peptides (ACPs) raised increasing interest in oncology research, as their obvious advantages as potential anti-cancer drugs.

ACPs consist of several cationic amino acids, which carry positive charge and display amphipathic structure, just in similarity with anti-microbial peptide [5]. Based on this, the primary action of ACPs is to damage cell membrane followed by cell lysis [6,7]. Additionally, triggering cell apoptosis through activating the mitochondria-mediated intrinsic pathway or the death receptor-mediated extrinsic pathway is also caused by some ACPs [8,9]. ACPs have attracted significant attention for cancer treatment because of their beneficial properties including being present in different sources, efficacy not restricted by drug resistance and have a great ability for the tissue penetration [10]. Furthermore, many ACPs display multiple cytotoxic effects in multidrug-resistant (MDR) cancer cells [11,12]. However, ACPs applied in cancer therapy still face challenges, in particular their reported instability under physiological conditions and the poor tumor-specific cytotoxicity [13]. Thus, some improvements are needed to promote peptide-based drugs into anti-cancer therapies.

Gene therapy technology was first discovered in 1972 and developed further with significant improvements in spite of various challenges [14,15]. For instance, 76.1% gene therapy clinical trials to 2017 in the world were carried out in the patients with solid or hematological malignancies [16]. Currently, a growing body of studies have reported that anti-cancer treatment options based on gene therapy can be effective in multiple malignant tumors including breast, ovarian, prostate, and lung cancers [17–20]. Some promoters have been used for tumoral gene therapy for their property of highly transcriptional activity in cancer cells but low or absence of activity in the normal cells [20,21]. Human Telomerase reverse transcriptase (hTERT) promoter has been reported to be activated in majority of cancer cells but not in normal cells as telomerase activity has been primarily detected in the malignant cells [22]. Thus, hTERT promoter is a preferential option for tumor gene therapy because of its specific tumor-targeting property and broad-spectrum activities in promoting tumorigenesis [23,24]. The efficiency of hTERT promoter-based gene therapy in cancers has been validated in previous studies [25–27]. Thus, in our hypothesis that ACP combined with hTERT promoter-based gene therapy would present promising tumor selective cytotoxicity.

Therefore, this study aimed to design novel ACPs and improve its tumor-specific cytotoxicity. The anti-cancer activity was discovered in many anti-microbial peptides, in this present study we used several anti-microbial peptide fragments, which have been shown to have anti-cancer activity [28–30], as basis and mimicked the characters of ACP to design some peptides by online tools. Among them, peptide KK-64 was selected out for the similar physicochemical properties as ACP and the significant anti-tumor activity against liver cancer cells. A gene deliver plasmid contained cancer-specific promoter hTERT was constructed to drive kk-64 gene specifically expressed in liver cancer cells. The recombinant plasmid pcTERT-kk-64 specifically inhibited liver cancer cells proliferation and induced apoptosis in vitro. Significant tumor regression was observed upon pcTERT-kk-64 plasmids treatment in tumor bearing mouse models. Furthermore, T cell infiltration was observed after pcTERT-kk-64 plasmid treatment. Our data provide evidence that the recombinant plasmid pcTERT-kk-64 is a promising cytotoxic drug candidate for live cancer.

## Materials and methods

### Reagents

High glucose Dulbecco’s Modified Eagle Medium (DMEM), RPMI-1640 Medium, Transwell insert and Matrigel were obtained from Corning (NYC, NY, USA). Bovine serum (BS) was purchased from Gibco (CA, USA). Penicillin, streptomycin, and 3′-Dioctadecyloxacarbocyanineperchlorate (DIO) were obtained from Sigma Aldrich (Louis, MO, USA). Opti-MEM medium, pcDNA3.1, Lipofectamine 2000 and TRIzol reagent were procured from Invitrogen (CA, USA). Restriction enzyme, DNA polymerase, dNTPs, T4 DNA ligase, and PrimeScript RT reagent kit were all purchased from TaKaRa (Dalian, Liaoning, China). PCR product purification kit and gel extraction kit were obtained from TIANGEN (Beijing, China). FastStart Universal SYBR Green Master (ROX), Annexin-V-FLUOS staining kit, and protein inhibitor cocktail tablets were purchased from Roche (Basel, Switzerland). CellTiter 96® Aqueous One Solution Cell Proliferation Assay (MTS) kit was obtained from Promega (Madison, WI, USA). ThermoFisher Scientific (MA, USA) provided the PierceTM BCA Protein Assay kit. Polyvinylidene Fluoride (PVDF) membranes were obtained from Merck (MA, USA). Bovine Serum Albumin (BSA) was purchased from Genview (FL, USA). ECL luminescent liquid was obtained from YEASEN (Shanghai, China). Cleaved caspase 3, Bcl-2, and Bax antibodies were purchased from Cell Signaling Technology (Danvers, MA, USA). β-Actin, cleaved caspase-8, and 9 antibodies were purchased from Abclonal (Wuhan, Hubei, China) PARP-1 antibody was obtained from ThermoFisher Scientific (MA, USA). CD3, CD8, CD45 antibodies, and HRP-conjugated secondary antibody were obtained from Santa Cruz (CA, USA). DAB luminescent liquid was purchased form MXB biotechnologies (Fujian, China). All other chemicals used were analytical reagent grade.

### Polypeptides

The bioactive fragments from Buforin II, CA-MA and Temporin anti-microbial peptides were used as basis to design peptides by Discovery studio software [31]and screened using viability assay against HepG2 cells. The molecular weight, isoelectric point, grand average of hydropathicity and instability index was calculated by ProtParam tool. The contents of α-helix and β-fold in the secondary structure were predicted in Network Protein Sequence@nalysis system based on Hybrid Neural Network (HNN). The hydrophobicity/hydrophilicity was evaluated by ProtScale tool. The optimal hybrid peptide referred as KK-64 and the amino acid sequence was as following:

MKKRLLRGRLLRIKKILSLIGGLLKWKLFKKIGIGKFLHSWKKFGGRWGLQFPVGRVHRLLRKK.

### Cell viability assay

HepG2 and HL-7702 cells were plated in 96 well plates at a density of 8 × 10^4^/well (HepG2) or 1 × 10^5^/well (HL-7702). The cells were then treated with KK-64 peptide at 2, 4, 6, 8, 10 μM for 24 h, and an equal volume of DMSO was added to the vehicle groups. After treatment, MTS reagent was added to the cells and the plates were incubated further for 1 h. Thereafter, the absorbance was determined at 490 nm using Biotek Microplate Reader (Vermont, USA). The IC50 values of KK-64 peptide in HepG2 or HL-7702 cells were calculated by SPSS Statistics V 17.0.

### Lactate dehydrogenase (LDH) release assay

To determine the LDH release of cells upon KK-64 treatment, HepG2 and HL-7702 cells were plated into 96 well plates at a density of 8 × 10^4^/well (HepG2) or 1 × 10^5^/well (HL-7702). 3.5 μM KK-64 peptides that has been diluted with DMEM containing 1% BS were added to the cells and incubated for 0.5, 1, 2, 4, 6 h, respectively. Thereafter, DMEM containing 1% BS and 1% Triton X-100 was added to the cells as a positive control group and the cells that were exposed to DMEM only containing 1% BS were used as a negative control. The supernatant of cells was collected at each time point, and the LDH content was determined by Cytotoxicity Detection Kit according to the manufacturer’s instruction. In brief, 100 μl of the samples were mixed with detection reagent in 1:1 ratio and incubated away from light at room temperature for 30 min. The absorbance was determined at 450 nm by Microplate Reader. The relative LDH release was calculated based on the formula:

$$\begin{array}{rl} & LDH\;release\;\left(\%\right)\;=\left(OD490\;of\;experimental\;group\;\right.\\ & \;\left.-\;OD490\;of\;negative\;group\right)/\left(OD490\;of\;positive\;group\right.\\ & \left.-\;OD490\;of\;negative\;group\right)\times 100\% \end{array}$$

### Construction of KK-64 expression plasmid

To obtain kk-64 DNA sequence, the KK-64 amino sequence was reverse-translated into DNA sequence using optimized codons for human and synthesized by GENEWIZ (Suzhou, Jiangsu, China). The pcTERT plasmid was constructed as described previously [32]. kk-64 DNA fragment was digested with *HindIII* and *XbaI* and subsequently sub-cloned into pcTERT to generate pcTERT-kk-64 plasmid.

### Cell culture and transfection

Human hepatocellular carcinoma cell line (HepG2) and mouse hepatoma carcinoma cell line H22 were obtained from the American Type Culture Collection (VA, USA). Human normal hepatocyte cell line HL-7702 was purchased from Type Culture Collection of the Chinese Academy of Sciences. HepG2 and HL-7702 cells were maintained in DMEM supplied with 10% (v/v) BS, 100 U/mL penicillin, and 100 μg/mL streptomycin, H22 cells were cultured with RPMI-1640 medium supplied with 10% (v/v) BS. All the cells were grown at 37°C, in an atmosphere of 5% CO_2_. No sign of mycoplasma contamination was observed in all cell lines.

HepG2 and HL-7702 cells were transiently transfected with indicated quality of pcTERT-kk-64 or pcTERT plasmids (100 ng in 96 well plate, 1.5 μg in 12 well plate, and 2.5 μg in 6 well plate) using lipofectamine 2000. Briefly, the plasmids and lipofectamine 2000 were diluted by opti-MEM, respectively, and incubated for 5 min; thereafter, the plasmids and lipofectamine were mixed and subsequently incubated further for 20 min. The mixture was then added to HepG2 and HL-7702 cells. 6 h later, the culture medium was replaced with fresh one and the culture was continued for further 64 h. The viability of both HepG2 and HL-7702 cells after plasmid transfection for 72 h was analyzed as described above.

### Analysis of apoptosis by flow cytometry

For quantification of apoptotic cell population, HepG2 and HL-7702 cells were seeded into 6 well plates at a density of 2 × 10^5^/well or 2.2 × 10^5^/well respectively. The cells were transfected with pcTERT-kk-64 or pcTERT plasmids and cultured for 72 h. Thereafter, the cells were trypsinized and stained with Annexin V-fluorescein and PI for 15 min at room temperature. After that, the cells were detected using flow cytometer (BD Bioscience, San Jose, CA, USA), and the data were analyzed using the CellQuest software (BD Bioscience, San Jose, CA, USA).

### Transwell migration and invasion assays

HepG2 cells were seeded in 12 well plates and transfected with pcTERT or pcTERT-kk-64 plasmids. After incubation for 48 h, the cells were trypsinized and diluted to 2.5 × 10^5^/ml with serum-free DMEM. To determine the ability of cell migration upon plasmid transfection, 200 μl cell suspension was plated into 8 μm pore size cell culture inserts and incubated in 300 μl DMEM containing 10% BS for 13 h. For detecting the cell invasion after plasmid transfection, Matrigel was thawed at 4 C and mixed with serum-free DMEM in a 1:8 ratio. 200 μl cell suspension was seeded in inserts that was coated with 100 μl Matrigel mixture and cultured in 300 μl complete medium for 19 h. After migration or invasion, the cells were fixed by using 4% paraformaldehyde for 30 min, and after that, the cells were stained with crystal violet for 30 min and cells in the upper chamber were wiped. Photographs of nine different fields were taken using a 100× magnification in optical microscope (Olympus, Tokyo, Japan) and migrated or invaded cells were counted by Image J software (USA).

### Cell membrane morphology observation

To observe the morphological changes in HepG2 and HL-7702 cells after plasmid transfection, the cells were plated on the glass slides, which were placed into 6 well plates, and then cells were transfected with pcTERT or pcTERT-kk-64 plasmids. After transfection for 72 h, the cells were fixed by 4% paraformaldehyde. DIO was used to stain the cell membrane, and the nucleus was stained by DAPI. The cell membrane morphology was observed using a confocal laser scanning microscopy (Olympus, Tokyo, Japan).

### Mouse experiments

6–8 weeks male BALB/c mice (weight 25 ± 0.21 g) were obtained from Huafukang Bioscience (Beijing, China) and maintained under 12 h light-12 h dark cycle with ad libitum access to water and food. All animal experiments were performed in accordance with the guidelines for the Care and Use of Experimental Animals of Jilin University and were approved by the Animal Experiment Ethics Committee of Jilin University. H22 hepatoma transplanted tumor model was constructed referred to previous study [33]. Briefly, two mice were inoculated with H22 cells by intraperitoneal injection and maintained for 10 days. The ascites was collected, and H22 cells were diluted to 1.5 × 10^7^/ml with saline solution. Each mouse was injected subcutaneously with 0.2 ml cell solution in the right fore axillary region. Nine days post inoculation, a clear tumor mass was observed. Twenty inoculated mice were randomly divided into two different groups (n = 10) for pcTERT or pcTERT-kk-64 plasmid administration. All the mice received 300 μl DNA:liposome complexes containing 100 μl Lipofectamine 2000 and 50 μg pcTERT or pcTERT-kk-64 endotoxin-free plasmids through intra-tumoral injection every 3 days for 9 days. The tumor size was measured every day and the tumor volume was calculate with the formula: Volume (cm^3^) = length (cm) × width^2^ (cm^2^). The mice were sacrificed 4 days after the last plasmids administration. The tumors were removed from the mice, and relative indexes were determined in the various experiments.

### Hematoxylin and eosin (H&E) staining and immunohistochemistry

Tumor specimens were isolated after mice have been sacrificed and fixed in 4% paraformaldehyde, embedded in paraffin wax, then 5-µm-thick paraffin-embedded sections were cut and stained with hematoxylin and eosin (H&E). For immunohistochemistry, paraffin-embedded sections were subjected to deparaffinization, rehydration, followed by antigen retrieval with 0.01 M sodium citrate. The sections were thereafter treated with 3% H_2_O_2_ for 10 min to inactivate any endogenous peroxidase and then incubated for 30 min in blocking buffer (5% bovine serum albumin in phosphate buffer Saline). Then the sections were incubated with mouse anti-CD3 antibody (1:300) or anti-CD8 (1:300) antibody or anti-CD45 (1:300) antibody overnight at 4, followed by incubation with HRP-conjugated secondary antibody (1:100) at room temperature. The Images were acquired using a microscope (Olympus, Japan), and quantitative analysis was carried by Image J (National Institutes of Health, USA) software using the IHC Profiler plugin [34], IHC score was calculated by the formula [35]: Score = (percentage contribution of high positive) × 4 + (percentage contribution of positive) × 3 + (percentage contribution of low positive) × 2 + (percentage contribution of negative) × 1/100.

### Western blot analysis

Transfected HepG2 and HL-7702 cells or tissues were lysed with lysis buffer (50 mM HEPES, 1% Triton X-100, 100 mM sodium fluoride, 50 mM sodium pyrophosphate, 10 mM sodium vanadate, 10 mM EDTA, protease inhibitor cocktail) and the concentration of protein samples were measured by BCA protein assay kit. The protein samples were separated by 12% SDS-PAGE gel electrophoresis and transferred onto the PVDF membrane. Subsequently, membrane was blocked with 2% BSA in TBST for 1 h and incubated with primary antibodies overnight at 4°C. The HRP-conjugated secondary antibodies were diluted with TBST and incubated further for 1 h at room temperature. The blots were thereafter imaged using ECL reagent on a Tanon 5200 Multi fluorochrome image system (Tanon, Shanghai, China).

### RNA extraction and quantitative real-time PCR (qRT-PCR)

Total RNA was extracted from transfected HepG2 cells or tissues using the TRIzol reagent according to the manufacturer’s protocols. RNA (1 μg) was reverse-transcribed into cDNA using PrimeScript RT reagent kit according to the manufacturer’s instructions. qRT-PCR was carried out with the cDNA as template and using FastStart universal SYBR green master (ROX) reagent. Thereafter, amplification and detection were conducted by StepOne plus real-time PCR machine (Applied Biosystems, Carlsbad, CA, USA). The relative gene expression level was analyzed using the 2-ΔΔCt method and normalized to GAPDH. The cycling conditions used were as follows: 95°C for 10 min, followed by 40 cycles of 95°C 15 s, 60°C 1 min. The specific primers sequences have been indicated below:

GAPDH (forward, 5ʹ- GCACCACCAACTGCTTAG-3ʹ; reverse, 5ʹ-GCAGGGATGATGTTCTGG-3ʹ)

MMP-2 (forward, 5ʹ- GATACCCCTTTGACGGTAAGGA-3ʹ; reverse, 5ʹ-CCTTCTCCCAAGGTCCATAGC −3ʹ)

MMP-9 (forward, 5ʹ- GACGCAGACATCGTCATCCA-3ʹ; reverse, 5ʹ-AACTCGTCATCGTCGAAATGG −3ʹ)

### Statistical analysis

All data has been shown as mean ± standard errors of the mean (SEM) and the statistical significance was analyzed by two-tailed unpaired student’s t-tests. *P* < 0.05 was accepted as statistically significant.

### Results

The current therapies can’t not treat patients with liver cancer satisficaly, thus, efficeint anti-tumor durgs is urgently needed to be developed. This present study was aimed to design novel ACPs, the potential anti-tumoral drug with obvious advantages, and explored their anti-tumoral effects in vitro and in vivo. We screened KK-64 peptide, which showed strong cytotoxicity to liver cells and cloned its DNA into recombinant plasmid which driven by cancer cell-specific promoter hTERT, the anti-tumor activity of recombinant plasmid pcTERT-kk-64 was tested in HepG2 cells and H22 transplanted mouse tumor model.

### Cytotoxicity of KK-64 peptide against liver cells

**Figure 1.**
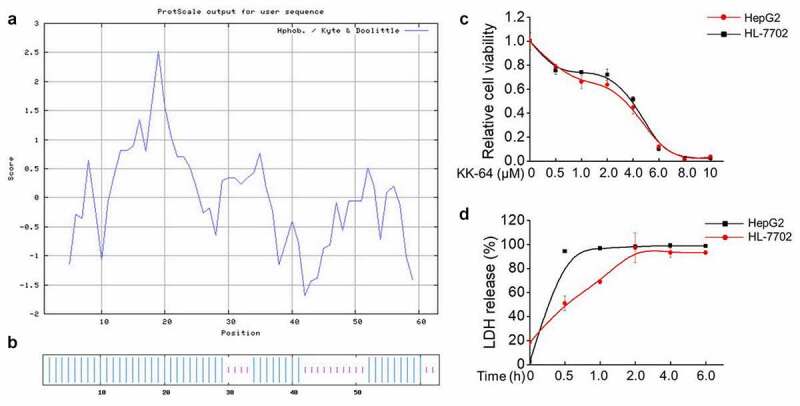
The novel ACP KK-64 showed significant cell killing effect. (a) The hydrophobicity/hydrophilic properties of KK-64 peptide were evaluated by ProtScaled tools. (b) The secondary structure of KK-64 peptide was predicted using Network protein Sequence@nalysis system based on Hybrid Neural Network (HNN). (c) The different concentrations of KK-64 peptide were added to HepG2 and HL-7702 cells, equal volume of DMSO was added as the vehicle group. The cell viability was determined by MTS assay. (d) The supernatant of HepG2 and HL-7702 cells were collected upon KK-64 peptide treatment for 0.5, 1, 2, 4, 6 h respectively. The LDH release was detected using cytotoxicity detection kit

To obtain efficient anti-cancer peptides (ACPs), bioactive fragments from the numerous anti-microbial peptides were combined and mimic synthesized into anti-tumor peptides using Discovery studio software. Finally, the fragments from Buforin II, CA-MA and Temporin anti-microbial peptides were optimally combined to generate a peptide, which consisted of 64 different amino acid residues. As shown in Table 1, the molecular weight of peptide, which referred as KK-64 was 7503.14 Da and the isoelectric point was as 12.85, thereby indicating that KK-64 was a cationic peptide. A − 0.232 hydrophobic index illustrated the hydrophilic nature of KK-64. To further verify the hydrophilicity of KK-64 peptide, the hydrophobicity/hydrophilic properties of KK-64 amino acid sequence was assessed by ProtScaled tools. The result showed a dominant hydrophilic domain in KK-64 peptide (Figure 1(a)). Instability index of KK-64 was 34.19 and less than 40, which indicated a predominantly stable nature of KK-64. The lipophilic character of KK-64 was verified by the 120.63 lipid solubility index (Table 1). Most of ACPs possess α-helix secondary structure that can render them superiority in degrading the cell membranes. The prediction result showed that KK-64 peptide contained 73.02% α-helix (Table 2, Figure 1(b)), which convincingly demonstrated its anti-tumoral property. Overall, the features of KK-64 peptide were found to be consistent with the reported characteristics of majority of ACPs.

**Table 1.** Physicochemical indexes of KK-64 peptide

MW	IP	GRAVY	Instability index	Aliphatic index
7503.14	12.85	−0.232	34.19	120.63

MW: molecular weight; IP: isoelectric point; GRAVY: Grand average of hydropathicity.

**Table 2.** The ratio of conformations in the secondary structure of KK-64 peptide

	Alpha-Helix	Coil
KK-64	73.02%	26.98%

The bioactivity of KK-64 peptide was studied against HepG2 and HL-7702 cells. It was observed that the viability of both of HepG2 and HL-7702 cells were significantly inhibited upon KK-64 peptide treatment in a dose-dependent manner (Figure 1(c)). The IC50 value of KK-64 peptide to HepG2 cells was 3.256 μM and to HL-7702 cells was 3.603 μM, which illustrated a strong cytotoxic potential of KK-64 peptide against liver cancer and normal liver cells. To further explore whether KK-64 peptide can potentially damage cell membrane, lactic dehydrogenase (LDH) release was detected, and the result showed that relative LDH release of HepG2 and HL-7702 cells reached maximum maximal value in an extremely short time after exposure to 3.5 μM KK-64 peptide. Moreover, LDH from HepG2 cells was released in 0.5 h, whereas HL-7702 cells released LDH reached the maximal level in 4 h (Figure 1(d)), thereby indicating a possible ability of KK-64 peptide to cause significant damage to the cell membrane. In general, KK-64 peptide appeared to possess excellent bioactivity against liver cells.

### Recombinant plasmid pcTERT-kk-64 selectively inhibits the growth and induces apoptosis of liver cancer cells

**Figure 2.**
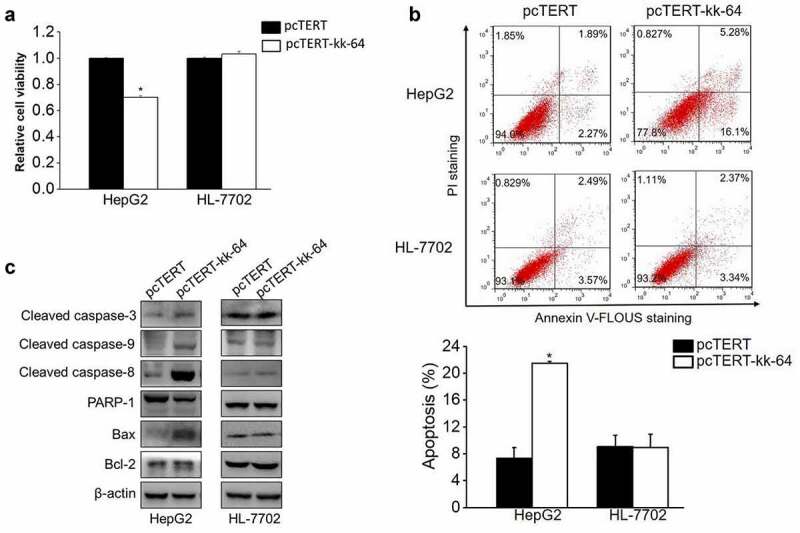
pcTERT-kk-64 plasmid selectively inhibit liver cancer cells growth and induced apoptosis. HepG2 and HL-7702 cells were transfected with pcTERT-kk-64 or pcTERT plasmid. The cell viability was tested using MTS kit (a) and the apoptosis was determined by Annexin-V-FLUOS/PI staining method using flow cytometer (b). Experiments were repeated three times. The levels of apoptosis regulating proteins were detected by Western blot analysis (C). Data represent as mean ± SEM, *, *P* < 0.05

The non-distinctive damage induced by KK-64 peptide on the liver cancer and normal liver cells can restrict the possible application of KK-64 peptide. To enhance the specificity of this peptide toward the tumor cells, kk-64 DNA sequence was inserted into hTERT promoter-based vector pcTERT. Transient transfection of pcTERT-kk-64 plasmids suppressed the growth rate in HepG2 cells by 30% as compared with that of those transfected with pcTERT plasmids only. Interestingly, pcTERT-kk-64 plasmids showed no significant cytotoxicity against HL-7702 cells (Figure 2(a)). This result demonstrated that pcTRET-kk-64 plasmids can specifically inhibit the growth of tumor cells while showing no significant killing effects on the normal cells.

To determine the effect of pcTERT-kk-64 plasmids on cellular apoptosis, HepG2 and HL-7702 cells were transfected with pcTERT-kk-64 or pcTERT plasmids. The results of flow cytometer showed that the apoptotic rate in pcTERT-kk-64 transfected HepG2 cells was increased by 14.15% as compared to that in pcTERT transfected HepG2 cells. However, there was no significant difference of apoptotic rate in HL-7702 cells, which were transfected with pcTERT-kk-64 or pcTERT plasmids (9.07 ± 1.7% vs 8.94 ± 1.98%) (Figure 2(b)). Several anti-apoptotic and pro-apoptotic genes can regulate and activate the process of apoptosis [36]. In pcTERT-kk-64 transfected HepG2 cells, caspase-9, caspase-8, and caspase −3 were cleaved that indicated their activation and the expression level of Bax was increased, however the protein level of total PARP-1 was found to be decreased (Figure 2(c)), which indicated that endogenous mitochondrial pathway and exogenous death receptor pathway were simultaneously activated and apoptosis was induced upon pcTERT-kk-64 plasmids transfection in HepG2 cells. In accordance with the result of flow cytometer, the expression of these pro-apoptotic factors was not affected in HL-7702 cells after transfection with pcTERT or pcTERT-kk-64 plasmids (Figure 2(c)). In conclusion, pcTERT-kk-64 plasmids can selectively trigger apoptosis in tumor cells but did not have a significant apoptotic effect on the normal cells.

### hTERT promoter driven kk-64 expression can significantly inhibit HepG2 cells migration and invasion

**Figure 3.**
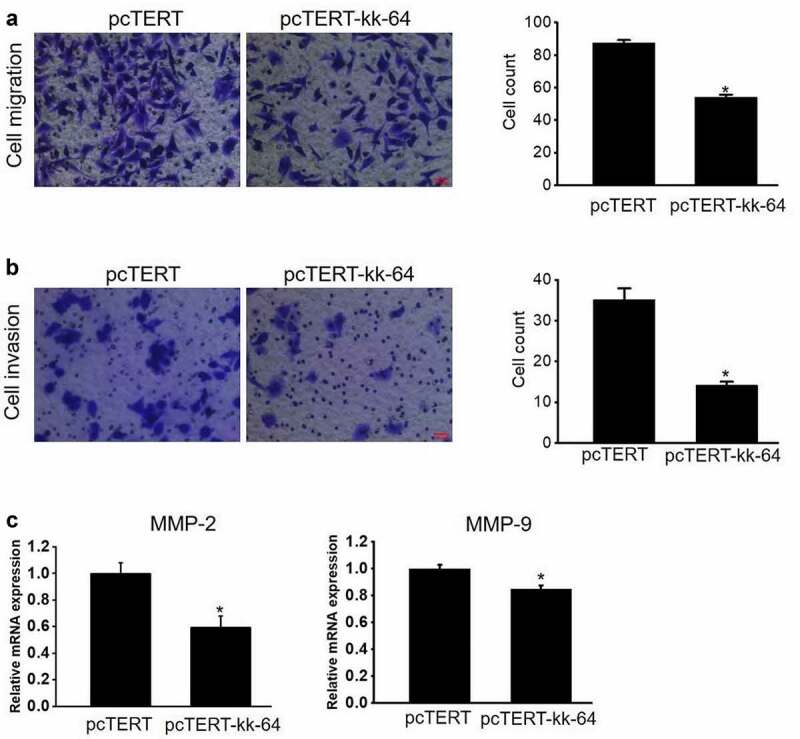
pcTERT-kk-64 plasmid can suppress migration and invasion of HepG2 cells. pcTERT-kk-64 or pcTERT plasmid were transfected in HepG2 cells. The cell migration (a) and invasion (b) was detected using transwell inserts and the number of migrated and invaded cells were counted in 9 different fields. Three replicates were performed in this assay. (c) The expression of MMP-2 and MMP-9 was determined by qRT-PCR analysis. Data are means ± SEM. *, *P* < 0.05

Since migration and invasion are two important hallmarks of cancer cells, hence experiments were performed to analyze the migration and invasion potential of HepG2 cells after pcTERT-kk-64 plasmids transfection. The results clearly showed that the cell counts of pcTERT-kk-64 plasmids transfected HepG2 cells undergoing migration and invasion were markedly less than that in pcTERT transfected groups (Figure 3(a-b)). Notably, the mRNA levels of MMP-2 and MMP-9, the genes reported to regulate cellular migration and invasion, were significantly decreased in pcTERT-kk-64 plasmids transfected HepG2 cells (Figure 3(c)). These results indicated that the expression of kk-64, which was driven by hTERT promoter can attenuate both the migration and invasion potential of HepG2 cells.

### pcTERT-kk-64 plasmids transfection can substantially damage the cell

**Figure 4.**
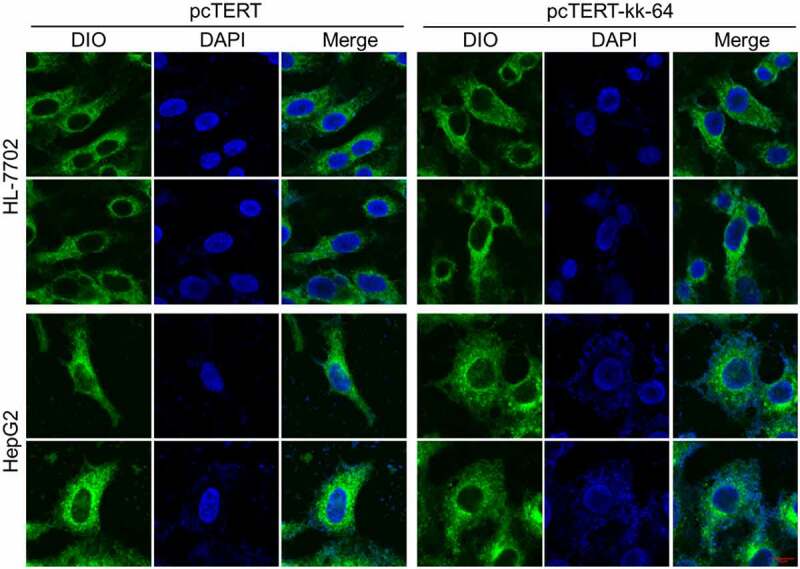
The membranes of HepG2 cells were damaged after pcTERT-kk-64 plasmid transfection. pcTERT-kk-64 or pcTERT plasmids were transfected into HepG2 and HL-7702 cells. The cell membrane was stained by DIO and the cell nuclei was stained by DAPI. The morphological changes in HepG2 cells and HL-7702 cells were observed using confocal laser scanning microscopy

As described above that KK-64 peptides possess the potential to damage the cell membrane, whether pcTERT-kk-64 plasmids transfection can damage the cell membrane was explored. The images showed that upon pcTERT-kk-64 plasmids transfection, HepG2 cell membranes were broken followed with nuclear leakage, and the morphology of cells was significantly altered as compared with cells that transfected with pcTERT plasmids (Figure 4). However, the morphology of HL-7702 cell membranes was noticed to be intact after transfection with pcTERT or pcTERT-kk-64 plasmids (Figure 4). The results illustrated that pcTERT-kk-64 plasmids also can effectively damage cell membranes and this destructive effect was specifically targeted toward the tumor cells.

### pcTERT-kk-64 plasmid represses tumor growth in vivo

**Figure 5.**
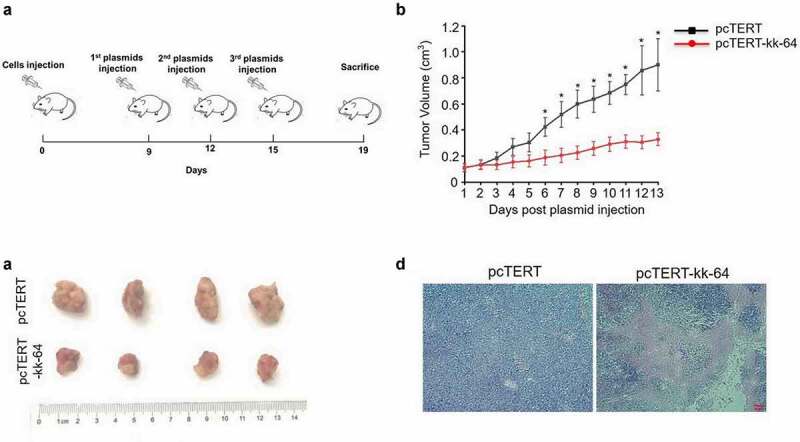
pcTERT-kk-64 plasmid inhibited H22 cell bearing tumor growth. H22 cell tumor–bearing syngeneic BALB/c mice were given plasmid–liposome complex containing pcTERT or pcTERT-kk-64 plasmid vial intra-tumoral injection. (a) Schematic diagram of processing time. (b) The tumor volume was detected everyday post plasmid injection (n = 10 for each group). (c) The Tumors were removed 4 days after the last administration of the plasmid. (d) The tissue specimens from the tumors were fixed and processed for H&E staining

To investigate the potential cytotoxicity of pcTERT-kk-64 plasmid in vivo, H22 cell tumor-bearing mice model was established, and pcTERT-kk-64 plasmid or pcTERT plasmid were injected for the treatment. As Figure 5 shows, the tumor volume gradually increased after injection and a significant tumor tissue mass was observed (Figure 5(a-b)). Notably, tumor volume was significantly reduced after pcTERT-kk-64 plasmid treatment as compared to pcTERT plasmid injected mice (Figure 5(a-b)). Furthermore, obviously necrocytosis was observed in the tumor tissues of pcTERT-kk-64 plasmid injected mice (Figure 5(c)). All these results indicated that pcTERT-kk-64 plasmid showed significant activity to retard the tumor growth in vivo.

### pcTERT-kk-64 plasmid triggered substantial apoptosis and immune cell infiltration in vivo

**Figure 6.**
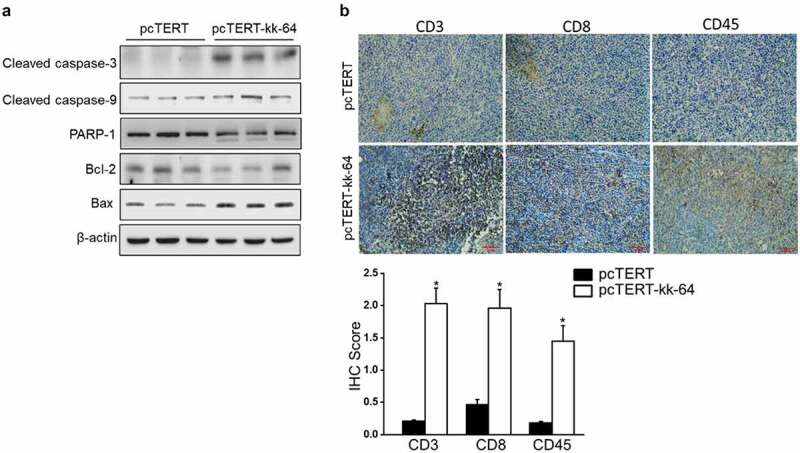
pcTERT-kk-64 plasmid triggered cell apoptosis and T cells infiltration in tumor tissue. (a) The changes in the expression of various apoptosis markers were determined by Western blot analysis in the tumor tissues. (b) The tumor specimens were fixed and immunohistochemical analysis of CD3, CD8 and CD45 was carried out, quantitative assessment of antibodies staining intensity was performed by Image J software (n = 5)

As pcTERT-kk-64 plasmid can induce apoptosis in HepG2 cells, the effect of pcTERT-kk-64 plasmid injection in causing apoptosis in the tumor tissues was analyzed. The results showed that cacpase-3 and caspase-9 levels were significantly activated after pcTERT-kk-64 treatment, whereas the expression of total PARP-1 and Bcl-2 was substantially reduced in pcTERT-kk-64 group. However, the expression of Bax protein was increased in pcTERT-kk-64 group (Figure 6(a)). These results indicated that pcTERT-kk-64 plasmid can promote cell apoptosis in the tumor tissues.

The CD3^+^, CD8^+^, and CD45^+^ T cells have been shown to be critical in eradicating tumor cells, extensive T cell infiltration promotes the formation of an antitumor microenvironment [37]. Hence, The expression of CD3, CD8, and CD45 was also analyzed in the tumor tissues. As indicated by immunohistochemistry results, there was a low-level expression of CD3, CD8, and CD45 in pcTERT group, while the expression of these markers was significantly increased after pcTERT-kk-63 plasmid treatment, and the IHC score was at least 1.5-fold higher than pcTERT group as quantitative analysis showed (Figure 6(b)), which indicated that pcTERT-kk-64 injection effectively promoted infiltration of cytotoxic T cell into the tumor tissues.

## Discussion

Peptides are easy to manipulate and have significantly low genotoxicity, which makes them an attractive candidate for anti-tumor drug development [38,39]. Although numerous natural peptides have been developed and even applied in clinical trials [40], some vital advantages related to synthetic peptides, such as the flexible modifiability, can potentially increase their use as novel therapeutic agents [41]. Thus, a novel anti-tumor peptide was designed and synthesized in this study. A number of prior reports have established that several potent anti-microbial peptides can also display significant anti-tumoral activities [42,43]. Therefore, KK-64 peptide was designed based on bioactive fragments of anti-microbial peptides. KK-64 peptide was optimally selected because of its excellent physicochemical properties as it was a hydrophilic cationic peptide, and the secondary structure was mainly composed of α-helix, which was in accordance with the characteristics displayed by commonly used anti-tumor peptides [44]. KK-64 exhibited significant cell killing effects against both HepG2 cells and HL-7702 cells. Notably, it caused a significant damage to the cell membranes in a short time. These results indicated that KK-64 was biologically active to a great extent, but this peptide did not display any specificity toward the tumor cells. Generally, the tumor cell membrane possesses electronegativity, and hence anti-tumor peptides designed to target them are usually cationic in nature [45]. Additionally, hydrophobic effects and greater fluidity of tumor cell membranes may also influence the selectivity of anti-tumor peptides [45]. The physicochemical properties of KK-64 peptide were predicted by bioinformatics tool, and it was noted that most of the observed characteristics can meet those exhibited by cancer-specific anti-tumoral peptides.

Some promoters have been extensively used in cancer-target gene therapy because of the specific activation in cancer cells and their limited activity in normal cells [46]. The significant potential of hTERT promoter in development of tumor-targeted therapies has been confirmed in previous studies [47–49]. In this paper, KK-64 DNA sequence was delivered to the cells and the tumor tissues by using a modified pcDNA3.1 plasmid named pcTERT-kk-64 and expressed under the control of hTERT promoter. Transient transfection with pcTERT-kk-64 plasmid could significantly inhibit the cell viability and induce substantial apoptosis of liver cancer cells but exhibited no pharmacological effects on the normal liver cells. These results suggested that KK-64 DNA sequence was selectively expressed in cancer cells and suppressed the growth of cancer cells in vitro. Moreover, transfection with pcTERT-kk-64 plasmid also significantly inhibited migration and invasion potential of HepG2 cells, which indicated that pcTERT-kk-64 plasmids can attenuate cancer progression. As KK-64 peptides could break cell membrane to damage the cells, the membrane morphology was also observed after transfection with pcTERT-kk-64 plasmid. It was noticed that the membranes of HepG2 cells were substantially damaged but no change was noticed in the membranes of HL-7702 cells. This result clearly illustrated that KK-64 was expressed in cells and can retain its membrane disrupting property. Furthermore, it was only expressed in cancer cells and could effectively damage the membranes in liver cancer cells.

The cell-based findings with pcTERT-kk-64 plasmid were also further validated in a pre-clinical model. Interestingly, pcTERT-kk-64 plasmid also displayed significant anti-cancer activity in vivo, as the tumor growth was significantly repressed after pcTERT-kk-64 plasmid injection. H&E staining showed a strong cytotoxicity of pcTERT-kk-64 plasmid against tumor cells, and apoptosis was triggered after pcTERT-kk-64 plasmid treatment. The increased expression of CD3, CD8, CD45 also indicated that pcTERT-kk-64 plasmid could effectively modulate immune response in the tumor tissues. Cytotoxic T cells exhibit potent anti-cancer effects against tumor tissues, and therefore, pcTERT-kk-64 plasmid demonstrated significant anti-tumor activity partly through activating anti-tumor immunity.

Although amounts of peptides, no matter natural or synthetic, were researched and reported to have anti-cancer activity in vitro and in vivo [50], the innovation of our study is combining the synthetic peptide with tumor selective promoter hTERT, the recombinant plasmid pcTERT-kk-64 have both cytotoxicity and tumoral selectivity. Furthermore, hTERT can be activated in many different types of cancer cells, the tumor suppressive actions of pcTERT-kk-64 may probably also apply to other cancer cells. Overall, pcTERT-kk-64 plasmid could possibly serve as a potential novel candidate for pharmacologically targeting aberrant tumor growth.

## Conclusion

The hTERT promoter driving KK-64 peptide expression showed tumor selective cytotoxicity in liver cancer cells. Furthermore, pcTERT-kk-64 plasmid inhibits mouse bearing hepatoma by triggering cell apoptosis and activating anti-tumoral immunity via infiltrating T cells.

## Data Availability

The datasets used in this study are available from the corresponding author upon reasonable request.

## Highlights

- KK-64 peptide shows strong cytotoxicity on liver cancer cells.
- pcTERT-kk-64 specific causes tumor regression.
- pcTERT-kk-64 activates anti-tumoral immunity via triggering T cell infiltration.

## Abbreviations

ACP: Anti-cancer peptide; BS: Bovine serum; DIO: 3′-Dioctadecyloxacarbocyan-ineperchlorate; DMEM: Dulbecco’s Modified Eagle Medium; GRAVY: Grand average of hydropathicity; H&E staining: Hematoxylin and Eosin staining; HNN: Hybrid Neural Network; hTERT: Human Telomerase reverse transcriptase; IP: isoelectric point; MW: molecular weight; LDH: Lactate dehydrogenase; MDR: Multidrug-resistant; qRT-PCR: Quantitative Real-time PCR; SEM: Standard errors of the mean.
